# Relation between sex hormones and leucocyte telomere length in men with idiopathic pulmonary fibrosis

**DOI:** 10.1111/resp.13871

**Published:** 2020-06-24

**Authors:** Chuling Fang, Hui Huang, Qian Zhang, Na Wang, Xiaoyan Jing, Jian Guo, Martin Ferianc, Zuojun Xu

**Affiliations:** ^1^ Department of Respiratory and Critical Medicine Peking Union Medical College Hospital, Chinese Academy of Medical Sciences and Peking Union Medical College Beijing China; ^2^ Electronic and Electrical Engineering Department University College London London UK

**Keywords:** androgen metabolism, idiopathic pulmonary fibrosis, leucocyte telomere length, polymorphism, sex hormone

## Abstract

**Background and objective:**

IPF is an ageing‐related lung disorder featuring progressive lung scarring. IPF patients are frequently identified with short telomeres but coding mutations in telomerase can only explain a minority of cases. Sex hormones regulate telomerase activity in vitro and levels of sex hormones are related to LTL. The objective of this study was to explore whether sex hormones were associated with LTL, whether they interacted with genetic variants in telomerase and whether polymorphisms in the exon of androgen metabolism genes were associated with plasma testosterone concentrations in male IPF patients.

**Methods:**

A case–control study was performed on 101 male IPF subjects and 51 age‐matched healthy controls. Early morning plasma sex hormones were quantified, and whole‐exome sequencing was used to identify rare protein‐altering variants of telomerase and SNP in the exon of androgen metabolism genes. LTL was analysed by PCR and expressed as a T/S ratio.

**Results:**

LTL, testosterone and DHT were decreased significantly in the IPF group. After adjustments for age and variant status in telomerase‐related genes, only testosterone was positively associated with LTL (*P* = 0.001). No significant interaction (*P* = 0.661) was observed between rare protein‐altering variants of telomerase and testosterone. No coding SNP in androgen metabolism genes were significantly associated with testosterone concentrations.

**Conclusion:**

Plasma testosterone is associated with LTL independent of age or rare protein‐altering variants of telomerase. No genetic variations of androgen‐related pathway genes are associated with androgen concentrations. Further studies are warranted to examine whether hormonal interventions might retard telomere loss in male IPF patients.

## Graphical abstract

In this case–control study, we explored the role of sex hormones in IPF. We found that plasma testosterone levels decreased in male patients with IPF, and plasma testosterone was positively associated with LTL. No coding variations of androgen‐related pathway genes were associated with androgen concentrations.

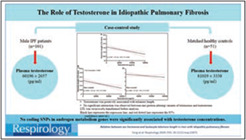

**Abbreviations**
AD: autosomal dominant
AR: autosomal recessive
BMI: body mass index
DHEA: dehydroepiandrosterone
DHT: 5α‐dihydrotestosterone
E2: oestradiol
FDR: false discovery rate
HRCT: high‐resolution computed tomography
IPF: idiopathic pulmonary fibrosis
LD: linkage disequilibrium
LTL: leucocyte telomere length
MAF: mutant allele frequency
PARN: poly(A)‐specific ribonuclease
PCR: polymerase chain reaction
PM: moderate pathogenicity
PP: supporting pathogenicity
PUMCH: Peking Union Medical College Hospital
PVS: very strong pathogenicity
RTEL1: regulator of telomere elongation helicase 1
SNP: single‐nucleotide polymorphism
SNV: single‐nucleotide variant
T: testosterone
T/S ratio: ratio of the copy number of telomere DNA to a single‐copy gene
TERC: telomerase RNA component
TERT: telomerase reverse transcriptase

## INTRODUCTION

Idiopathic pulmonary fibrosis (IPF) is a chronic, progressive and fibrotic interstitial lung disease of unknown aetiology, with a median survival of 2–3 years after the diagnosis.^1^ IPF occurs primarily in men aged from 50 to 70 years and its annual incidence increases dramatically with age.^2^ The level and role of sex hormones in IPF are almost unknown.

Telomeres are DNA–protein structures that are located at the ends of linear chromosomes and whose function is to protect chromosome ends from recombination and degradation.^3^ By synthesizing new telomeres, telomerase solves the ‘end‐replication problem’—successive shortening of telomeres with cell division.^4^ Telomere length is regarded as an important biomarker of ageing, and some earlier studies indicate that in pulmonary fibrosis patients the leucocyte telomere length (LTL) is shorter than in age‐matched normal controls.^5^ Rare protein‐altering variants in telomerase‐related genes (*TERT* (telomerase reverse transcriptase), *PARN* (poly(A)‐specific ribonuclease), *TERC* (telomerase RNA component) and *RTEL1* (regulator of telomere elongation helicase 1)) are shown to affect LTL in sporadic^6^ and familial pulmonary fibrosis.^7^, ^8^ However, coding mutations in telomerase cannot explain short LTL of all IPF patients with short telomere lengths.^5^

**Fig 1:**
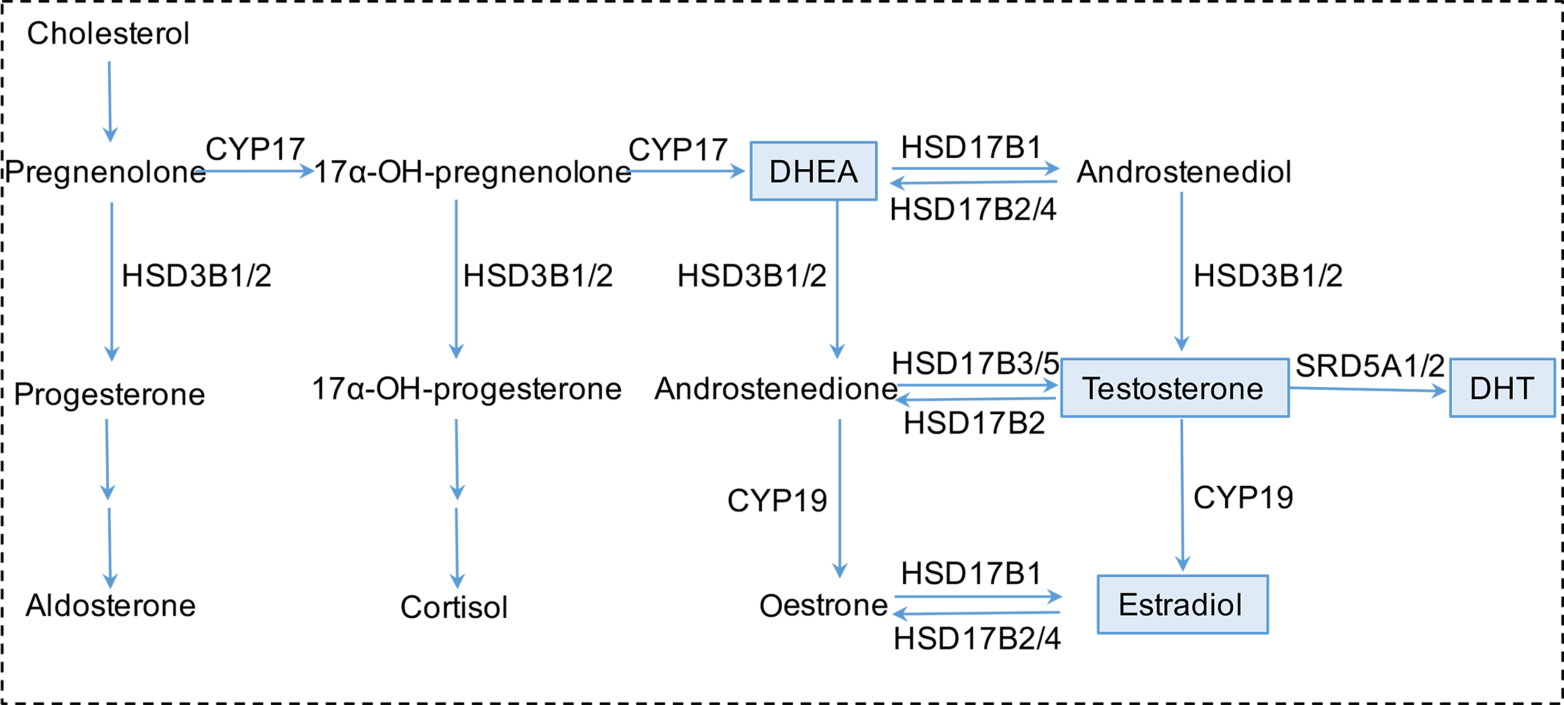
Androgen biosynthesis and metabolism pathway and genes involved in the pathway. DHEA, dehydroepiandrosterone; DHT, 5α‐dihydrotestosterone.

Considerable experimental evidence indicates that sex hormones regulate telomerase activity.^9^, ^10^, ^11^, ^12^, ^13^, ^14^, ^15^ Sex hormones are related to ageing and two metabolites of testosterone, 5α‐dihydrotestosterone (DHT) and oestradiol (E2), are found positively correlated with LTL in men.^16^ Testosterone/oestradiol ratio is lower in older men with coronary artery disease (CAD) and it is also significantly correlated with LTL.^17^ Moreover, the plasma levels of the precursor of testosterone (T), dehydroepiandrosterone (DHEA), are significantly decreased in male IPF patients.^18^ To date, clinical research on sex hormones and their associations with LTL in IPF patients has been limited. In addition, polymorphisms in androgen metabolism genes are found to be associated with sex hormone concentrations in men.^16^, ^19^ The androgen biosynthesis, metabolism pathway and genes involved in the pathway are shown in Fig. 1. T is metabolized by 5α‐reductase (encoded by *SRD5A1/2*) to DHT and by aromatase (encoded by *CYP19*) to E2.^16^, ^19^ In our study, we first investigated the level of sex hormones and then we explored the correlations of plasma sex hormones with LTL, and their interaction with rare protein‐altering variants in telomerase‐related genes (*TERT*, *PARN*, *TERC* and *RTEL1*). Finally, we explored the relationship of single‐nucleotide polymorphisms (SNP) in the exon of androgen metabolism genes with plasma T.

## METHODS

### Study design and study population

A case–control study on 101 male IPF subjects and 51 age‐matched controls was conducted in Peking Union Medical College Hospital (PUMCH) of the Chinese Academy of Medical Sciences and Peking Union Medical College (CAMS and PUMC), Dongcheng District, China. Participants were enrolled consecutively. The diagnosis of IPF was in accordance with the American Thoracic Society/European Respiratory Society/Japanese Respiratory Society/Latin American Thoracic Association (ATS/ERS/JRS/ALAT) consensus statement from 2018.^20^ Clinical and biopsy findings, and HRCT scans of each patient were independently reviewed by two experienced pulmonologists and one radiologist. All IPF subjects were Han Chinese and the IPF subjects were excluded if comorbidities including hypertension, diabetics, cardiovascular disease, endocrine disorders or malignancy occurred before the enrolment. The heathy subjects were recruited from the Health Screening Center of PUMCH. Inclusion criteria for controls selection were as follows: (i) male, age‐matched and smoking status matched; (ii) Han Chinese; and (iii) exclusion of hypertension, diabetics, cardiovascular disease, endocrine disorders or malignancy. This study was approved by the Regional Ethics Committee of PUMCH (JS‐1127/2016) and written consent was obtained from all participating men. For each subject, demographic information, medical history, family history and other baseline information were also collected.

### Sex hormone measurements

Peripheral blood samples were collected from all enrolled subjects. Plasma samples were produced by centrifugation and subsequently stored at −80°C until analysis. Plasma DHEA, T, DHT and E2 were measured by an enzyme‐linked immunosorbent assay (ELISA) using commercially available kits (Novus Biologicals, Centennial, CO, USA).

### LTL assay

Telomere lengths of leucocyte DNA samples were determined by real‐time quantitative polymerase chain reaction (PCR) in StepOne Plus real‐time PCR system (Applied Biosystems, Foster City, CA, USA), using the protocol previously described by Cawthon.^21^ Both the telomere and single‐copy control gene (β‐globin) of each sample were amplified in triplicate and the median value was used for the analyses. The ratio of the copy number of telomere DNA to a single‐copy gene (T/S ratio) was normalized to a reference sample of the control group in this study. The relative T/S ratio reflected the average telomere repeat copy number of each DNA sample calculated relative to the reference DNA.^5^, ^22^ Twelve samples randomly chosen from included subjects were analysed to assess the reproducibility of the assay (Fig. S1 in Supplementary Information). For telomere and β‐globin PCR, the overall coefficient of variance (CV) was 2.7 ± 1.4% and 2.0 ± 1.4%, respectively.

### Analysis of variants of telomerase and androgen metabolism genes

Whole‐exome sequencing was performed to identify coding variants of telomerase and androgen metabolism genes. Extraction of DNA was performed following standard procedures using the QIAamp Genomic DNA mini kit (QIAGEN, CA, USA). A minimum of 1 μg of DNA per sample was used for the DNA library generation. First, genomic DNA samples were randomly fragmented by sonication to an average size of 180–250 bp. Second, the DNA fragments were end‐repaired, A‐tailed and ligated to adapters, followed by PCR amplification. Lastly, the DNA libraries were sequenced on Illumina HiSeq X platform (Illumina Inc., San Diego, CA, USA) with a paired‐end read length of 150 bp (PE150).

Whole‐exome sequencing reads were mapped to GRCh37/hg19, using Burrows‐Wheeler Aligner (BWA) software to generate original BAM files.^23^ Then, these BAM files were sorted and aligned by SAMtools to compute the sequence coverage and depth.^24^ Single‐nucleotide variants (SNV) and insertions and deletions (indels) were called with GATK^25^ and annotated by ANNOVAR.^26^ The following filters were set to identify candidate variants: (i) remove mutations with coverage less than 10×; (ii) in the telomerase‐related genes (*TERT*, *PARN*, *TERC* and *RTEL1*), include variants with mutant allele frequency (MAF) <1% in the 1000 Genomes databases and the functional predictions by SIFT, PolyPhen‐2 and MutationTaster all indicated the SNV was not benign or if it had a high impact (e.g. stop gain, stop loss and frameshift); and (iii) in terms of genes involved in androgen metabolism pathway (*SRD5A2*, *CYP19A1*, *HSD3B2*, *HSD17B1*, *HSD17B2*, *HSD17B3*, *HSD17B4* and *AKR1C3*), no rare damaging protein‐altering variants were found in this study, and only common coding variant with a MAF of 5% or more was included in the analysis. All SNP were in accordance with Hardy–Weinberg equilibrium in the control group (*P* > 0.05, *P*‐value was corrected by false discovery rate (FDR)).

### Statistical analysis

Statistical analysis was performed using SPSS software version 24.0 for Windows (SPSS Inc., Chicago, IL, USA) and R statistical software (version 3.51, https://cran.r‐project.org/). Two‐tailed *P* < 0.05 was considered statistically significant and FDR was adopted to control the effects of multi‐testing. Non‐normal distribution values such as LTL and DHT were reciprocally transformed and DHEA was natural log‐transformed for the analysis. Results of continuous variables were reported as mean ± SD or median (interquartile range), while categorical variables were reported as a number with percentage. Comparison of basic data between two groups was done using Student's t‐test for continuous variables which fulfilled homogeneity of variance and using chi‐square test for categorical variables. Comparison of sex hormones levels between two groups was performed by multivariate analysis in the general linear model (GLM) with the adjustment of age. Correlations between sex hormones and LTL were analysed by partial correlation analysis to control confounding factors. The association of sex hormones with LTL was investigated using linear regression model, adjusted for age and variant status of telomerase. Subjects with rare coding variant of four telomerase‐related genes were assigned to ‘rare variant+’ group and those without mutations were in ‘rare variant−' group. This variant was included into the linear regression model as a dummy variant and its interaction with testosterone was also tested. The association of these SNP with T levels was determined using linear regression adjusted for age and case/control status. For all SNP analyses, the minor alleles were compared to the reference (the major allele homozygote). Linkage disequilibrium (LD) was investigated using the 1000G Phase‐3 population data in Haploview. SNP would be considered to be in a strong LD if r^2^ was greater than 0.8.^27^

## RESULTS

### Subject characteristics

A total of 101 male IPF patients and 51 matched controls were included in the study. Characteristics of 152 participants are summarized in Table 1. Cases and controls were similar in age and smoking status due to matching. No significant difference was found in BMI between the two groups (*P* = 0.431).

**Table 1:** Baseline characteristics of the included subjects

Characteristics	IPF cases (*n* = 101)	Controls (*n* = 51)	*P*‐value
Age	63.33 ± 8.11	63.22 ± 8.38	0.937
BMI (kg/m^2^)	24.11 ± 2.56	24.45 ± 2.53	0.431
Smoking status (%)			0.871
Former/current	39 (38.6)	19 (37.3)	
Never	62 (61.4)	32 (62.7)	
Clinical manifestation			
Cough (%)	98 (97.0)	0	
Dyspnoea (%)	76 (75.2)	0	
Finger clubbing (%)	49 (48.5)	0	
Velcro rales (%)	93 (92.1)	0	
PFT			
FVC (% predicted)	72.25 ± 15.21		
DL_CO_ (% predicted)	46.22 ± 12.76		

All values are reported as mean ± SD or percentage.

DL_CO_ % predicted, percent predicted diffusion capacity for carbon monoxide; FVC % predicted, percent predicted forced vital capacity; IPF, idiopathic pulmonary fibrosis; PFT, pulmonary function test.

### Sex hormones plasma levels

Male IPF subjects had significantly lower concentrations of T and DHT than healthy matched controls (*P* < 0.001). The levels of DHEA and E2 appeared to be lower in the IPF group than in the control group, but they did not reach statistically significant difference (*P* > 0.05) (Table 2).

**Table 2:** Plasma levels of sex hormones in men

	IPF cases (*n* = 101)	Controls (*n* = 51)	*P*‐value^†^
DHEA^‡^ (pg/mL)	2380.95 (1622.46–3652.33)	2716.79 (1871.41–4079.22)	0.638
T (pg/mL)	60 196.06 ± 2656.53	81 018.75 ± 3338.33	<0.001
DHT^‡^ (pg/mL)	382.05 (292.81–514.12)	530.44 (428.32–613.72)	<0.001
E2 (pg/mL)	377.09 ± 28.79	418.89 ± 33.05	0.375

All values are reported as mean ± SD or median (interquartile range).

† *P*‐value was calculated from general linear model adjusted for age.

‡ DHT was reciprocally transformed and DHEA was ln‐transformed before analysis.

DHEA, dehydroepiandrosterone; DHT, 5α‐dihydrotestosterone; E2, oestradiol; IPF, idiopathic pulmonary fibrosis; T, testosterone.

### LTL and rare variant frequency in telomerase‐related genes

Telomere length, expressed as a T/S ratio, was significantly shorter in male IPF subjects than in healthy controls (*P* < 0.001). Six IPF patients had a rare deleterious variant in *TERT*, one IPF patient had a rare variant in *PARN* and four in *RTEL1*, while no one in the control group had a deleterious variant in those genes. In total, rare coding variants in *TERT*, *TERC*, *PARN* and *RTEL1* were observed in 11 patients (10.9%) in the IPF group while none was found in the control group (Table 3). The detailed information of these rare deleterious variants is shown in Table 4.

**Table 3:** LTL and frequency of rare variants in telomerase genes in IPF patients and controls

	IPF cases (*n* = 101)	Controls (*n* = 51)	*P*‐value^†^
LTL^‡^	0.61 (0.49, 0.81)	1.16 (1.00, 1.38)	<0.001
Rare variants in telomerase complex component genes, *n* (%)			
TERT	6	0	
TERC	0	0	
PARN	1	0	
RTEL1	4	0	
Total	11 (10.9)	0	

All values are reported as mean ± SD or number.

† *P*‐value was calculated from general linear model adjusted for age.

‡ Variable was reciprocally transformed before statistical analysis.

IPF, idiopathic pulmonary fibrosis; LTL, leucocyte telomere length; PARN, poly(A)‐specific ribonuclease; RTEL1, regulator of telomere elongation helicase 1; TERC, telomerase RNA component; TERT, telomerase reverse transcriptase.

**Table 4:** Rare variants in telomerase‐related genes identified in 101 IPF cases

Patient	Gene	OMIM	Chr: position GRCh37/hg19 (rs refsnp)	1000g2015aug_all	HGVS identifier (protein)	Effect	Literature phenotype	SIFT	PolyPhen2_HDIV	MutationTaster	InterVar and_Evidence	Telomere length (T/S)
F7	RTEL1	608833	20:62364537G>C (rs778531697)	Absent	NM_001283010: c.G2224C:p.E742Q	Nonsynonymous	Dyskeratosis congenita AR/pulmonary fibrosis	Deleterious	Possibly damaging	Probably disease causing	Uncertain significance (PM1; PM2)	1.22
F10 and F54	TERT	187270	5:1279465 G>A	Absent	NM_001193376: c.C2071T:p.R691C	Nonsynonymous	Dyskeratosis congenita AD/pulmonary fibrosis	Deleterious	Probably damaging	Probably disease causing	Uncertain significance (PM1; PM2; PP3)	0.89; 0.63
F44	TERT	187270	5:1258758G>A	Absent	NM_001193376: c.C2798T:p.T933M	Nonsynonymous	Dyskeratosis congenita AD/pulmonary fibrosis	Deleterious	Probably damaging	Probably disease causing	Uncertain significance (PM1; PM2; PP3)	0.30
F60	TERT	187270	5:1293555G>−	Absent	—	Frameshift deletion	Dyskeratosis congenita AD/pulmonary fibrosis	—	—	—	Likely pathogenic (PVS1; PM2)	0.48
F66	PARN	604212	16:14704629_14704632 TAAC>−	Absent	—	Frameshift deletion	Dyskeratosis congenita AR/pulmonary fibrosis	—	—	—	Likely pathogenic (PVS1; PM2)	0.56
F77	RTEL1	608833	20: 62298873A>G (rs373656372)	Absent	NM_001283009: c.A666G:p.I222M	Nonsynonymous	Dyskeratosis congenita AR/pulmonary fibrosis	Deleterious	Probably damaging	Probably disease causing	Uncertain significance (PM1; PM2; PP3)	0.72
F85	TERT	187 270	5: 1266648A>T	Absent	NM_001193376: c.T2585A:p.L862Q	Nonsynonymous	Dyskeratosis congenita AD/pulmonary fibrosis	Deleterious	Probably damaging	Probably disease causing	Uncertain significance (PM1; PM2; PP3)	0.61
F86	TERT	187270	5: 1280332G>A	Absent	NM_001193376: c.C1891T:p.R631W	Nonsynonymous	Dyskeratosis congenita AD/pulmonary fibrosis	Deleterious	Probably damaging	Probably disease causing	Uncertain significance (PM1; PM2; PP3)	0.44
F88	RTEL1	608833	20: 62321484G>A (rs777153220)	Absent	NM_001283010: c.G1517A:p.R506H	Nonsynonymous	Dyskeratosis congenita AR/pulmonary fibrosis	Deleterious	Probably damaging	Probably disease causing	Uncertain significance (PM1; PM2; PP3)	0.54
F96	RTEL1	608833	20: 62324513C>T (rs398123018)	Absent	NM_001283010: c.C2200T:p.R734W	Nonsynonymous	Dyskeratosis congenita AR/pulmonary fibrosis	Deleterious	Probably damaging	Probably disease causing	Uncertain significance (PM1; PM2; PP5)	0.42

Refsnp (rs) single‐nucleotide polymorphism identifiers are provided where available.

1000g2015aug_all, the 1000 Genomes Project database; AD, autosomal dominant; AR, autosomal recessive; HGVS, Human Genome Variation Society; IPF, idiopathic pulmonary fibrosis; OMIM, Online Mendelian Inheritance in Man database; PARN, poly(A)‐specific ribonuclease; PM, moderate pathogenicity; PolyPhen‐2, Polymorphism Phenotyping version 2; PP, supporting pathogenicity; PVS, very strong pathogenicity; RTEL1, regulator of telomere elongation helicase 1; T/S ratio, ratio of the copy number of telomere DNA to a single‐copy gene; TERT, telomerase reverse transcriptase.

### Association between sex hormones and LTL

Next, we explored the role of sex hormones in IPF. Sex hormones were reported to regulate telomerase activity^9^, ^10^, ^11^ and their levels were associated with LTL.^16^, ^17^ Therefore, their relationship was investigated.

**Fig 2:**
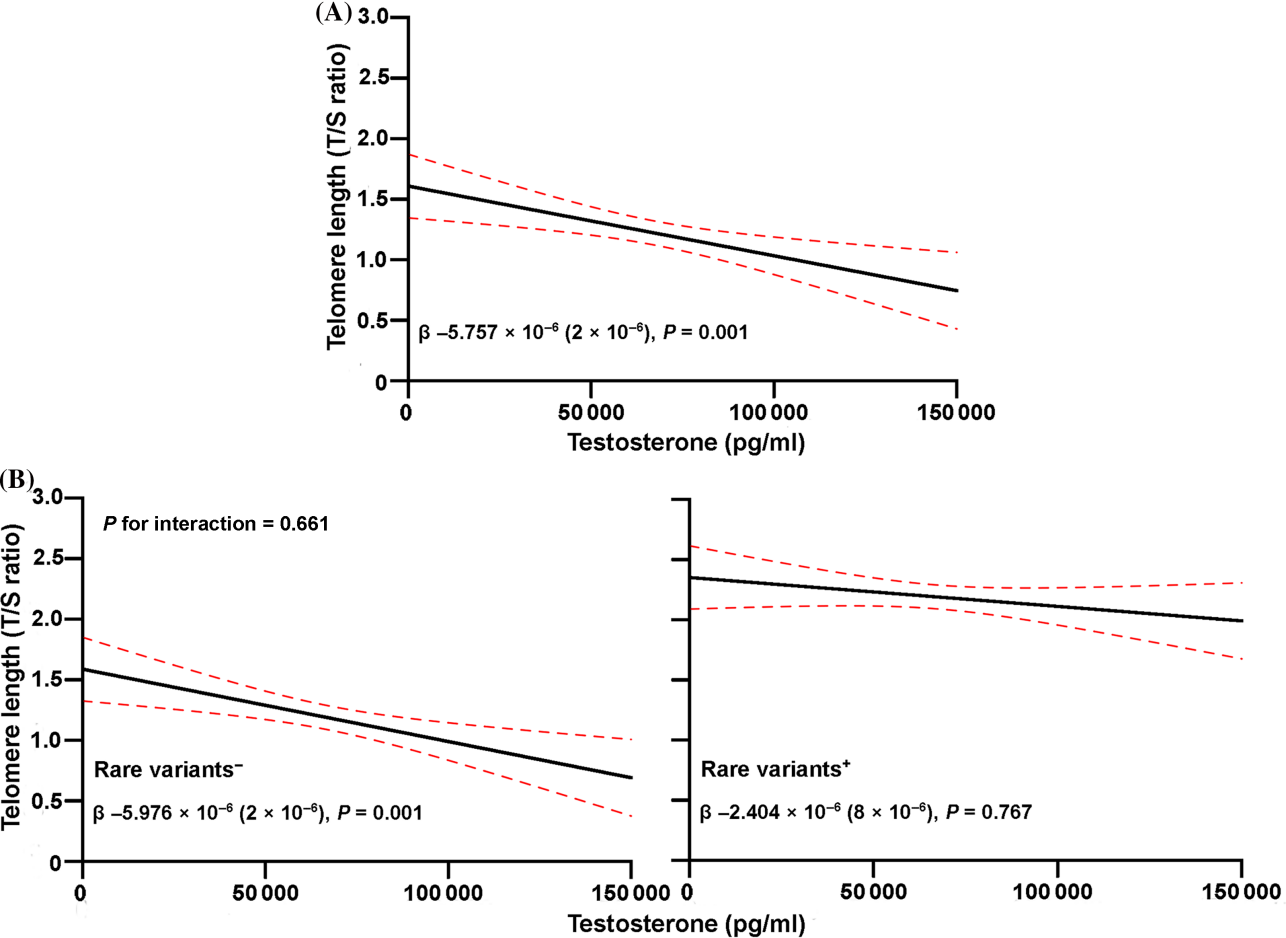
Potential association of testosterone with LTL in all included subjects (A), and interaction between rare variants in telomerase genes and testosterone on telomere length (B). LTL was reciprocally transformed before analysis. X‐axis represents the plasma level of testosterone (pg/mL) and y‐axis represents the reciprocal transformation of LTL (T/S ratio). Black line represents the regression line and red dashed line represents the 95% CI. ‘Rare variant+’ represents those subjects with rare coding variant in telomerase genes and ‘rare variant−’ refers to those without rare variants. (A) *P*‐value was adjusted for age and variant status in telomerase genes. (B) *P*‐value for interaction was listed and all the *P*‐values were adjusted for age. LTL, leucocyte telomere length; T/S ratio, ratio of the copy number of telomere DNA to a single‐copy gene.

In the partial correlation analyses (Table 5), levels of T and DHT were positively correlated with LTL after age adjustment (all *P* < 0.05), while no significant association was found between other sex hormones and LTL (all *P* > 0.05). In addition, after the adjustment for age, positive correlation was found between testosterone and its precursor (DHEA) and its metabolite (DHT) (all *P* < 0.05). Then, the associations of T and DHT with LTL were further evaluated by stepwise linear regression, and only T was significantly associated with reciprocal transformation of LTL after adjusting for age and variant status in telomerase‐related genes (β = −5.757 × 10^−6^ (2 × 10^−6^), *P* = 0.001) (Fig. 2A). Next, as the negative effects of rare protein‐altering variants in telomerase‐related genes (*TERT*, *PARN*, *TERC*, and *RTEL1*) on LTL were reported in the previous studies,^6^, ^28^ we investigated a potential interaction between T levels and rare variant status in telomerase and their effects on LTL. In the linear regression analyses after adjusting for age, no significant interaction was observed between T and rare variant status in telomerase (*P* = 0.661; Fig. 2B). It was obvious that LTL in subjects without rare variants in the telomerase‐related genes were positively associated with T levels (*P* = 0.001), while no significant association was found in individuals with rare variants (*P* = 0.767) (Fig. 2B).

**Table 5:** Partial correlation coefficients between sex hormones and telomere length as well as partial correlation coefficients between T and other sex hormones in male subjects

Variable	LTL^†^ (T/S ratio)		T (pg/mL)	
	Coefficients	*P* ^‡^	Coefficients	*P* ^‡^
DHEA^†^ (pg/mL)	0.105	0.200	0.268	0.001
T (pg/mL)	−0.231	0.004	—	—
DHT^†^ (pg/mL)	0.176	0.030	−0.688	<0.001
E2 (pg/mL)	−0.132	0.107	0.081	0.321

† LTL and DHT were reciprocally transformed and DHEA was ln‐transformed before analysis.

‡ *P*‐value was calculated from partial correlation analysis with adjustment of age.

DHEA, dehydroepiandrosterone; DHT, 5α‐dihydrotestosterone; E2, oestradiol; LTL, leucocyte telomere length; T, testosterone; T/S ratio, ratio of the copy number of telomere DNA to a single‐copy gene.

### Association between SNP of androgen metabolism genes and plasma androgen

A total of 13 SNP with MAF > 5% in the exon of genes involved in androgen biosynthesis and metabolism pathways were included for association analyses. In *AKR1C3*, SNP rs12529 was strongly correlated with rs12387 in East Asian population (r^2^ = 0.959).

Table 6 shows the associations between SNP in androgen metabolism genes and mean plasma T of all included subjects. In regression models adjusted for age and case/control status, no SNP was significantly associated with the T level (all *P*‐FDR > 0.05).

**Table 6:** Associations of SNP in the coding region of androgen metabolizing genes with testosterone concentration

Gene	SNP	Genotype	*n*	Mean	P‐FDR
SRD5A2	rs523349	GG	47	64 668.21 ± 4200.64	0.796
		GC + CC	105	68 308.12 ± 2634.82	
CYP19A1	rs700518	TT	51	69 283.03 ± 3705.29	0.786
		TC + CC	101	66 122.02 ± 2797.47	
	rs700519	GG	108	66 668.51 ± 2722.85	0.796
		GA + AA	44	68 444.54 ± 3888.97	
HSD17B1	rs605059	GG	47	59 810.56 ± 3802.96	0.786
		GA + AA	105	70 482.50 ± 2696.88	
HSD17B2	rs8191246	AA	143	66 803.54 ± 2301.05	0.796
		AG + AA	9	73 205.79 ± 9159.39	
HSD17B3	rs2066479	CC	96	69 663.80 ± 3031.45	0.796
		CT + TT	56	62 929.16 ± 3067.13	
	rs2066480	CC	134	67 600.46 ± 2392.88	0.796
		CT + TT	18	64 072.00 ± 6299.86	
	rs2066478	CC	133	67 840.44 ± 2398.79	0.796
		CT + TT	19	62 577.91 ± 6143.51	
HSD17B4	rs28943592	CC	122	66 539.21 ± 2491.58	0.796
		CT + TT	30	69 799.17 ± 5084.44	
	rs11205	AA	53	67 473.28 ± 3937.77	0.817
		AG + GG	99	67 027.01 ± 2717.50	
	rs25640	GG	38	65 036.18 ± 4716.81	0.796
		GA + AA	114	67 898.10 ± 2536.40	
AKR1C3	rs12529	GG	127	65 781.44 ± 2458.52	0.796
		CG + CC	25	74 300.60 ± 5188.37	
	rs12387	AA	130	65 781.88 ± 2402.24	0.892
		GA + GG	22	75 459.72 ± 5857.39	

Analyses were adjusted for age and case/control status.

P‐FDR, *P*‐value was corrected by false discovery rate; SNP, single‐nucleotide polymorphism.

## DISCUSSION

In this study consisting of 101 male IPF patients and 51 matched controls, significantly shorter LTL was observed in the IPF group in comparison to the matched healthy control group. However, only 10.9% of IPF patients showed rare protein‐altering variants in the exon of telomerase‐related genes, compared with none in the healthy controls. Shorter telomere in IPF patients was also confirmed in previous studies.^5^, ^29^ Meanwhile, a similar proportion of IPF patients (9% and 11.3%, respectively) with at least one rare variant in telomerase‐related genes (*TERT*, *PARN*, *TERC* or *RTEL1*) were reported.^6^, ^28^

IPF patients are primarily elderly men. The level of sex hormones in IPF is almost unknown. To date, only one earlier study by Mendoza‐Milla *et al*.^18^ in 2013 found that plasma DHEA, or its sulphated form, DHEA‐S was significantly and disproportionately decreased in male IPF patients and this adrenal steroid showed multiple antifibrotic properties in vitro. This study was the first to investigate the plasma levels of T, its precursor (DHEA) and metabolites (DHT and E2) in male IPF patients. We found that IPF patients had significantly lower plasma levels of T and DHT than the matched healthy controls.

Next, we focused on the role of sex hormones. A previous study by Huang *et al*.^17^ found that older men (≥60 years) with CAD had lower ratio of T to E2 (T/E2) and T/E2 was positively correlated with LTL. In another study among 980 men aged 17–90 years (7.7% of men with diabetes, 19.8% with hypertension and 20% with cardiovascular disease), positive correlations of two metabolites of T (DHT and E2) with LTL were reported.^16^ Anabolic androgenic hormones have been used to treat marrow failure syndrome since the 1960s.^30^, ^31^ In a clinical trial involving 27 patients with telomere diseases, treatment with the synthetic sex hormone danazol led to telomere elongation.^32^ In addition, considerable evidence from experiments indicated that androgens regulate telomerase^9^, ^10^, ^11^, ^12^ and treatment with androgens led to telomere elongation in a mouse model of telomere dysfunction.^33^ In our study, the associations between sex hormones and LTL were explored. Plasma T was significantly associated with LTL independent of age and also independent of variant status in telomerase‐related genes. We did not find any evidence that association between plasma T and LTL was modified by rare variants in telomerase‐related genes. These findings imply that in addition to rare variants in telomerase complex genes, lower T might be a potential factor accounting for shorter LTL in male IPF patients. It is possible that hormonal interventions might delay or reverse telomere loss in male IPF patients but has yet to be examined in sufficient detail.

Next, we explored the factors related to testosterone levels. Several previous studies evaluated the association of polymorphisms in genes involved in androgen biosynthesis and metabolism pathways with sex hormone levels in men.^16^, ^19^, ^34^, ^35^, ^36^, ^37^ Among them, one study reported that the minor allele of *SRD5A2* rs2208532 was associated with higher level of T in prostate cancer patients,^34^ while two other studies revealed that the minor alleles of SNP in *SRD5A2* (rs824811), *HSD17B1* (rs12602084), *HSD17B2* (rs1424151) and *HSD17B3* (rs9409407) reduced T concentrations in men with prostate cancer.^19^, ^37^ Similarly, in one research of 980 community‐dwelling men aged 17–90 years, the dominant allele of *SRD5A2* rs9282858 significantly increased T level while the dominant allele of *CYP19A1* rs17703883 was associated with decreased T concentration.^16^ Most of these SNP are in the intron or upstream regions of the genes and their functional activity remains to be determined.^19^, ^37^ However, in another study in which the relationship between 874 SNP in 37 candidate genes in the sex steroid hormone pathway and circulating T level was examined, none of the SNP in *SRD5A2* or *CYP19A1* were significantly associated with T level in Caucasian men.^35^ Similarly, in our study, no SNP in the exon of androgen metabolism genes were significantly associated with plasma T level. Given that only SNP in the exons were evaluated in our study, it might be that SNP in other regions of these genes would be appropriate for more detailed investigation.

We also present strengths and limitations to our study. This study was the first to evaluate the levels of sex hormones in male IPF patients and their relationship with LTL, as well as to explore associations between SNP in androgen metabolism genes and T levels. Blood samples were taken in the morning to minimize the effects of circadian variations on hormone levels. In addition, *P*‐value was corrected by FDR in terms of multiple tests, which was not achieved in most previous studies.^16^, ^19^ However, there are still several limitations to be aware of. First, only male IPF patients and matched controls were included in this study so our findings might not apply to women. The levels of sex hormones and their associations with LTL in female IPF patients should be examined in the future. Second, SNP associations in this study mainly focused on polymorphisms in the exons of genes involved in the androgen biosynthesis and metabolism pathway. Third, all blood samples were collected at the same time point, and we did not have continuous blood samples to investigate longitudinal changes in sex hormones levels or LTL. Lastly, a relationship was found between LTL and testosterone, but it is difficult to infer causality due to the nature of this study design. Further prospective studies and experimental studies are required to establish their links and explore their mechanism.

In conclusion, compared with matched controls, LTL, plasma level of T and DHT were significantly decreased in male IPF patients. Plasma T was associated with LTL independentl of age and rare protein‐altering variants in telomerase‐related genes. No genetic variations in the exons of androgen‐related pathway genes were associated with the T level. Our study suggests that lower level of T might play a role in the shorter LTL in male IPF patients apart from rare variants in telomerase‐related genes. Future research is needed to examine whether hormonal interventions might retard telomere loss in male IPF patients and whether such retardation is associated with clinically important outcomes.

## Author contributions

Conceptualization: C.F., H.H., Z.X. Data curation: C.F., H.H., Q.Z., N.W., X.J., M.F. Formal analysis: C.F., M.F. Project administration: Z.X. Methodology: C.F., H.H., J.G. Resources: H.H., Z.X. Visualization: Q.Z., N.W., X.J. Supervision: Z.X. Software: C.F. Writing—original draft: C.F. Writing—review and editing: C.F., H.H., Q.Z., N.W., X.J., J.G., M.F., Z.X.

## Supporting information


**Figure S1.** Reproducibility of average telomere length measurement by real‐time quantitative polymerase chain reaction.

**Visual Abstract** The role of testosterone in idiopathic pulmonary fibrosis.
