# Supplementary material for: Relation between sex hormones and leucocyte telomere length in men with idiopathic pulmonary fibrosis
Source: Respirology. 2020 Jun 24;25(12):1265–73. doi: 10.1111/resp.13871 (PMC7754418; doi:10.1111/resp.13871)
Supplement: Supplementary file 1 — Figure S1. Reproducibility of average telomere length measurement by real‐time quantitative polymerase chain reaction. [file RESP-25-1265-s002.docx]

**SUPPLEMENTARY INFORMATION**

**Relation between sex hormones and leukocyte telomere length in men with idiopathic pulmonary fibrosis**

Chuling Fang^1^, Hui Huang^1^, Qian Zhang^1^, Na Wang^1^, Xiaoyan Jing^1^, Jian Guo^1^,

Martin Ferianc^2^, Zuojun Xu^1^

^1^Department of Respiratory Medicine, Peking Union Medical College Hospital, Chinese Academy of Medical Sciences & Peking Union Medical College, Beijing, China.

^2^Electronic and Electrical Engineering Department, University College London, London, UK.

**Figure S1**: Reproducibility of average telomere length measurement by real-time quantitative PCR


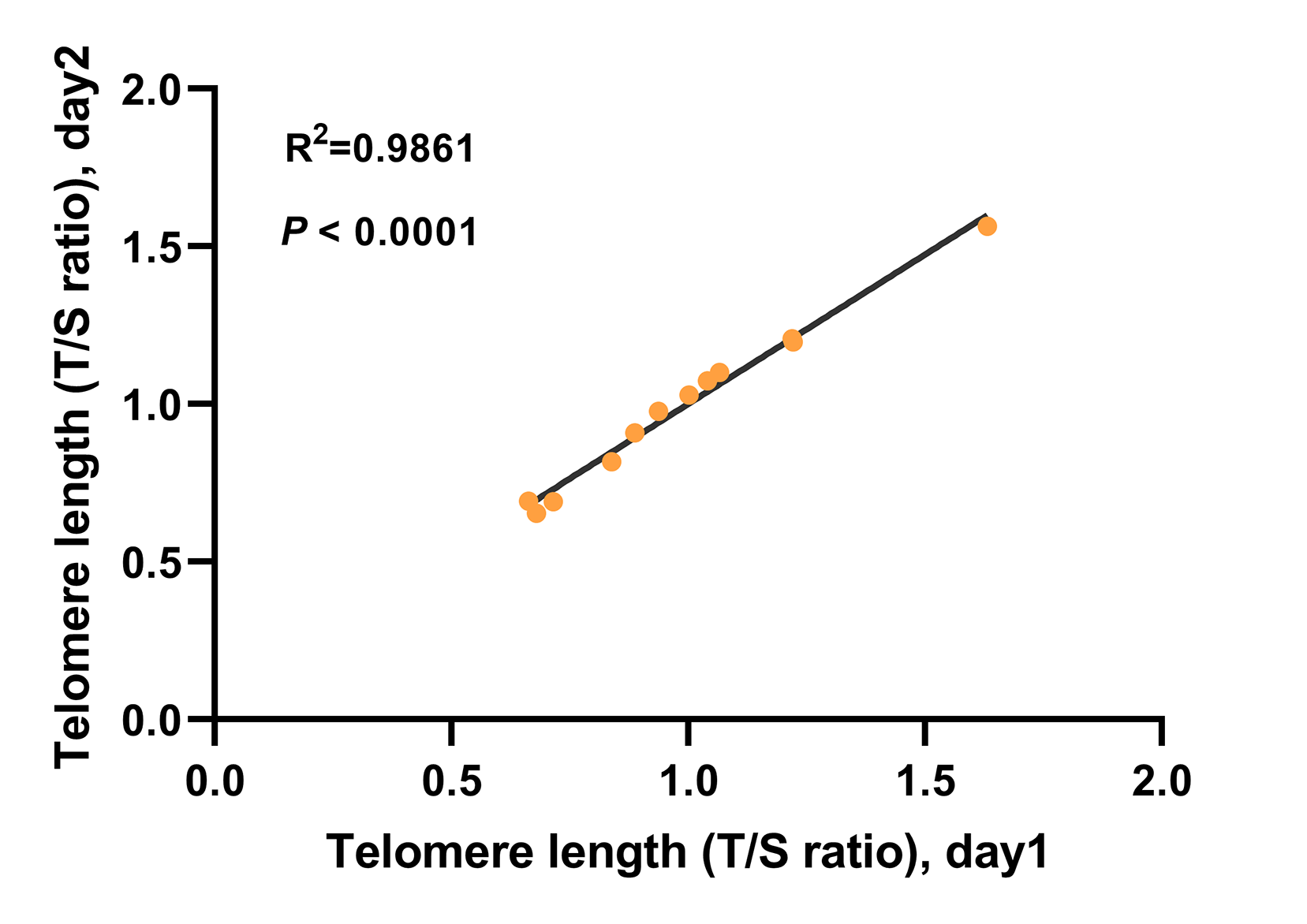


12 samples were tested in triplicate on consecutive days. A significant correlation was seen (R^2^=0·9861, p<0·0001).
