# Supplementary figures and images for: Relation between sex hormones and leucocyte telomere length in men with idiopathic pulmonary fibrosis
Source: Respirology. 2020 Jun 24;25(12):1265–73. doi: 10.1111/resp.13871 (PMC7754418; doi:10.1111/resp.13871)

## Slide 1
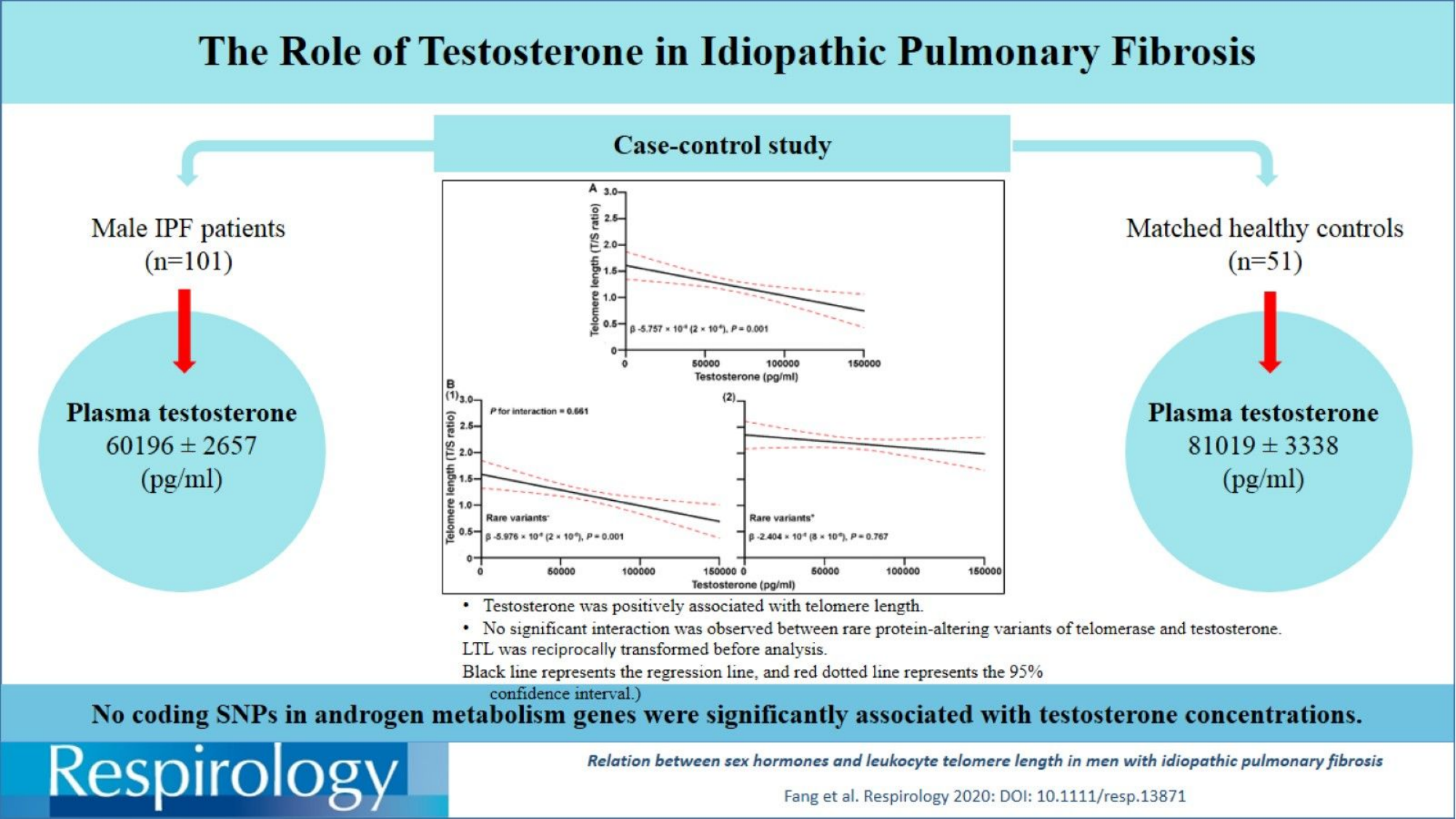

# The Role of Testosterone in Idiopathic Pulmonary Fibrosis

Supplement: Supplementary file 2 — Visual Abstract The role of testosterone in idiopathic pulmonary fibrosis. [file RESP-25-1265-s001.pptx]
